# Assessment of the cytotoxic effect of carboxymethyl chitosan-loaded amygdalin nanoparticles against human normal and cancer cell lines

**DOI:** 10.1186/s11671-024-03998-7

**Published:** 2024-04-04

**Authors:** A. S. El-Houssiny, A. A. F. Soliman, K. N. Abdel-Nour

**Affiliations:** 1 Microwave Physics and Dielectrics Department, National Research Centre, Dokki, Cairo Egypt; 2 Pharmacognosy Department, National Research Centre, Dokki, Cairo Egypt

**Keywords:** Carboxymethyl chitosan, Malignant cells, Amygdalin, Nanoparticles, Anticancer

## Abstract

In recent years, the development of antitumor drugs has been dedicated to natural products. Amygdalin is a natural herbal cyanoglycoside that has anticarcinogenic effect on many types of cancers once hydrogen cyanide (HCN) is released. The main objective of the present study is to synthesize and investigate the potential of carboxymethyl chitosan nanoparticles (CMC NPs) as drug delivery agents for amygdalin encapsulation and its delivery to cancer and normal cell lines. In this study, carboxymethyl chitosan nanoparticles encapsulated with amygdalin (CMC-Am NPs) were prepared and characterized through their particle size, surface charge, chemical structure and dielectric properties. Also, the *invitro* drug release of amygdalin from CMC NPs was studied. Additionally, the cytotoxcity of the amygdalin and CMC-loaded amygdalin NPs were evaluated through MTT assay. The results showed that the prepared CMC-loaded amygdalin NPs exhibited a small particle size of 129 nm, high zeta potential value of − 43 mV and confirmed the amygdalin stability and compatibility with CMC NPs. Furthermore, the CMC NPs demonstrated sustained release of amygdalin during 24 h. Moreover, compared to free amygdalin, amygdalin-loaded CMC NPs have significant anti-cancerous effect on human colon HCT-116 and breast MCF-7 cancer cell lines while being safe on normal cells BJ1. In conclusion, CMC NPs can be employed as an efficient drug delivery vehicle for controlled and sustained amygdalin release with enhanced cytotoxicity on malignant cells without harming normal cells.

## Introduction

Amygdalin, often known as vitamin B17, is an aromatic cyanogenic glycoside that is found in almond, peach, and apricot seeds [1]. It consists of one molecule of benzaldehyde, one molecule of hydrocyanic acid and two molecules of glucose. Amygdalin has been widely utilized as a natural anticancer medication because of the presence of cyanogenic group in its structure, which makes it an effective alternative for traditional cancer treatments [2]. The cancer cell contains enzyme beta-glucosidases which release the benzaldehyde and hydrogen cyanide (HCN) from amygdalin, which is a toxic compound. The cyanide ion decomposing carcinogenic cells by interfering with oxygen utilized by these cells and blocking the nutrient source of tumor cells [3].

Many experiments supported that the amygdalin has potential cytotoxic and anti-tumor effects and can be considered as a potential alternative for anticancer drugs [4–7]. Makarević et al. [8, 9] found that amygdalin reduced tumor cell proliferation in bladder and prostate cancer cell lines resulting in a significant delay in cell cycle progression. Ibrahim et al. [10] found that the apricot juice has an anticancer effect against colon cancer by reducing colon cancer biomarkers TNF-α, NF-κB, IL-1β and IL6 levels in the serum. Arshi et al. [11] observed that the amygdalin decreased growth and proliferation of stomach cancer (AGS), lung cancer (A549) and breast cancer (MCF7) through down-regulating the expression level of two antiapoptotic genes.

Various targeted therapeutic techniques, such as nanoformulation, have been developed to protect healthy cells from hydrogen cyanide and enhance amygdalin's anti-cancer properties [12]. Carboxymethyl chitosan (CMC) is a water-soluble derivative of chitosan which exhibits gel formation, water solubility, low toxicity as well as high biocompatibility that makes it a promising drug delivery system [13]. Due to the unique characteristics of CMC, various herbal and chemotherapy medicines have been encapsulated in CMC NPs in order to improve drug solubility, stability, achieve sustained release, minimize side effects, and inhibit tumor growth [14, 15].

Until now, amygdalin has been utilized alone, and few research have studied its administration via nanocarriers as anticancer. Additionally, amygdalin hasn’t been encapsulated yet with drug delivery nanocarriers such as carboxymethyl chitosan. To the best of our knowledge, this is the first study to focus on the utilization of amygdalin-loaded carboxymethyl chitosan nanoparticles as anticancer therapeutic agent for controlled drug delivery. In this work, we showed that encapsulating amygdalin in a nanoparticulate delivery system didn’t cause any toxicity to the skin fibroblast normal cells (BJ1), while significantly improving its anticancer therapeutic impact in the cancer cell lines of the breast (MCF-7) and colon (HCT-116). These findings could have a substantial impact on the use of CMC NPs to enhance amygdalin's therapeutic benefits in the treatment of cancer.

## Materials and methods

### Materials

Amygdalin (98% purity) was supplied by Roth (Germany). Carboxymethyl chitosan (CMC) was supplied by Santa Cruz Biotechnology (USA). Colon cancer cells (HCT-116), breast cancer cells (MCF-7) and skin normal fibroblast cells (BJ1) were purchased from karolinska Institute, Oncology and Pathology department, Stockholm.

### Preparation of CMC-Amygdalin NPs

Amygdalin loaded-CMC NPs were synthesized by the ionic-gelation method with CaCl_2_ as a cross linker [16]. CMC solution was prepared by dissolving it in distilled water and stirred for one hour at room temperature. Then, amygdalin was prepared in distilled water and added to CMC solution at a weight ratio (1:1) and stirred for 24 h. After that, CaCl_2_ (1%) was dropped into a CMC solution that had been preincubated with amygdalin at a 1:0.8 CMC: CaCl_2_ weight ratio. The NPs were obtained after 1.5 h of continuous stirring at room temperature.

## Characterization of amygdalin and amygdalin loaded-CMC NPs

### Particle size analysis and Zeta potential measurements

The average diameter, size distribution, and zeta potentials of amygdalin, CMC NPs as well as amygdalin-loaded CMC NPs were determined using a particle size analyzer (Malvern Instruments- Nano-ZS, Ltd., UK). The samples were sonicated for 3 min just before assessment. The NPs were measured in an aqueous suspension three times to calculate the average value.

### Attenuated total reflection-Fourier transform infrared (ATR–FTIR)

The possible interaction between amygdalin and CMC NPs was determined using ATR–FTIR spectroscopy. Amygdalin, CMC NPs as well as amygdalin-loaded CMC NPs FTIR spectra were obtained using an ATR-FTIR spectrophotometer Bruker VERTEX 80 (Germany), combined with a Platinum Diamond ATR disc as an internal reflector. The samples were placed into the ATR crystal, and the FTIR spectra were acquired with a resolution of 4 cm^−1^ in the scanning range of 400–4000 cm^−1^.

### Dielectric spectroscopy

Using an impedance analyzer (Schlumberger Ltd.-Solartron 1260, UK), the dielectric constant (ε') and dielectric loss (ε'') values of the amygdalin, CMC NPs, as well as amygdalin-loaded CMC NPs were measured at room temperature over a broad frequency range between 0.1 Hz and 5 MHz. The dielectric measurements were made on the samples in the form of solid pellets using a hydraulic press. The dielectric analysis was made by joining the impedance analyzer instrument to a pc via a GPIB IEE488 cable. For data acquisition, a commercial interfacing and automation program Lab VIEW was used. The temperature was adjusted by a temperature regulator equipped with a Pt100 sensor. The percentage of error in ε'' and ε' are 3% and 1%, respectively.

### *InVitro* drug release study

The dialysis assay was used to determine the release of amygdalin from CMC NPs. The dialysis tube (MWCO: 14,000 Da) was first cut, and then it was immersed in distilled water to fully hydrate it. After that, CMC-Am NPs (280 mg) was incubated into the dialysis tube and suspended in a beaker of 100 ml release medium (phosphate buffer solution) at pH 7.4. The medium was stirred using a magnetic stirrer at 400 rpm and 37°C. Two milliliters of the sample were removed from the release medium at predetermined intervals and replaced with an equivalent volume of fresh buffer. The amount of amygdalin released by CMC NPs at a given time was measured using UV–visible spectrophotometer at 262 nm.

### *In- Vitro* anticancer activity studies (MTT assay)

The MTT assay, a colorimetric test based on the ability of live cells to selectively convert the tetrazolium component of MTT to purple colored formazan crystals was used to measure the cytotoxic activity. Colon cancer cells (HCT-116), breast cancer cells (MCF-7) and skin normal fibroblast cells (BJ1) were cultured in DMEM-F12 medium under 5% CO_2_ and 37 ºC. Trypsin was used to separate the cells from the flask and they were subsequently resuspended in the growth medium. After that, using a water jacketed Carbon dioxide incubator, cells were seeded at density of 10^4^ cells/well on 96-well plates at 37 ºC and 5% CO_2_. Subsequently, the cancer cells were cultured for 48 h at varying doses of amygdalin and CMC-Am NPs (40, 20, 10 mg/ml). The media containing the cells without the addition of the samples were used as negative control. Doxorubicin, a recognized cytotoxic natural substance, was employed as positive control and yields 100% lethality. MTT was added to each group after 48 h of incubation, and then the cells were incubated for another 4 h. Following the formation of formazan crystals, sodium dodecyl sulphate (SDS) was used to dissolve them. An Elisa reader (model 3350, Bio-Rad Laboratories Inc., USA) was used to measure the absorbance at 595 nm. For each concentration, the samples were tested three times and each experiment was performed three times. An independent program t-test SPSS11 was used to examine the statistical significance between samples and negative control.

## Results and discussion

### Characterization of amygdalin and CMC-Am nanoparticles

#### Particle size and zeta potential measurements

The NPs characteristics like size and surface charge can influence on their uptake by living cells. The surface area of nanoparticles is large due to their small particle size, which allowing for strong adhesion to cells and faster drug release. This strong adhesion indicates a greater chance that the NPs can be internalized by cells [17].

**Fig. 1:**
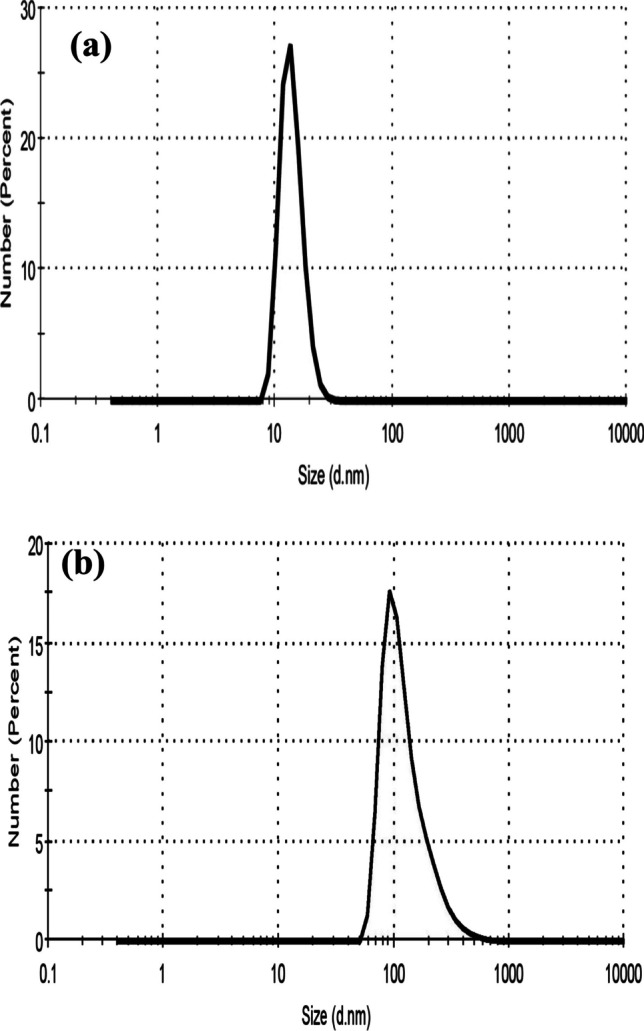
Particle size of **a** CMC NPs (15 nm) and **b** CMC-Am NPs (129 nm)

*Particle size analyzer* was used to measure the average size of blank CMC NPs as well as CMC-loaded amygdalin NPs as illustrated in Fig. 1. According to Fig. 1a, it was found that blank CMC NPs have an average diameter of 15 nm. Therefore, the current nano-formulation resulted in a small nano-size in the range of 100 nm. The encapsulation of amygdalin within CMC NPs caused an increase in size to 129 nm; Fig. 1b. Therefore, the CMC-loaded amygdalin NPs has a particle size larger than blank CMC NPs, hence indicated that amygdalin was successfully encapsulated within NPs. Furthermore, the PDI values for CMC NPs and CMC-Am NPs were found to be 0.503 and 0.263, respectively. These small PDI values indicate homogenous size distribution of NPs and low affinity to form aggregates.

**Fig. 2:**
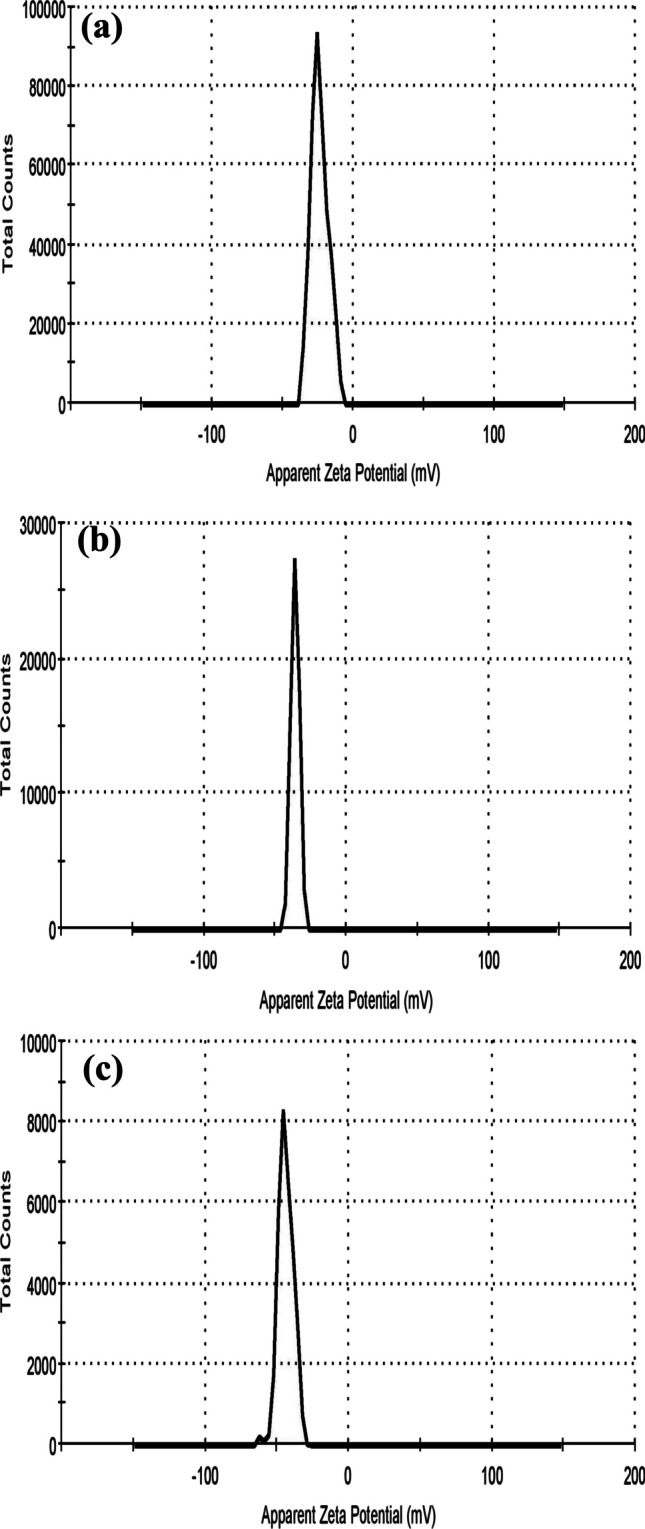
Zeta potential of **a** amygdalin (− 23 mV), **b** CMC NPs (− 35.8 mV) and **c** CMC-Am NPs (− 43.8 mV)

*Zeta potential* is a crucial characterization method that estimates the surface charge of NPs and therefore predicts their colloidal stability. Nanoparticles which have zeta potentials larger than positive 30 mV or more negative than—30 mV are highly stable because the charges on the surface inhibit the aggregation of the particles [18]. Figure 2a indicates that amygdalin has a negative zeta potential value of − 23 mV. According to Fig. 2b, CMC NPs have a negative zeta potential value of − 35.8 mV [19]. After the encapsulation of amygdalin within CMC NPs, the zeta potential value slightly increased to − 43.8 mV as illustrated in Fig. 2c. Thus, amygdalin encapsulation slightly alters the NPs surface charge [20]. The large zeta potential value observed for CMC NPs before and after amygdalin encapsulation indicated the excellent stability of the prepared nano-formulations. The excessively anionic charged carboxyl groups on the NPs surface reduce their aggregations due to the strong repelling forces between the particles in suspension and leads to a stable dispersion of NPs.

#### Attenuated total reflection-Fourier transform infrared (ATR–FTIR)

**Fig. 3:**
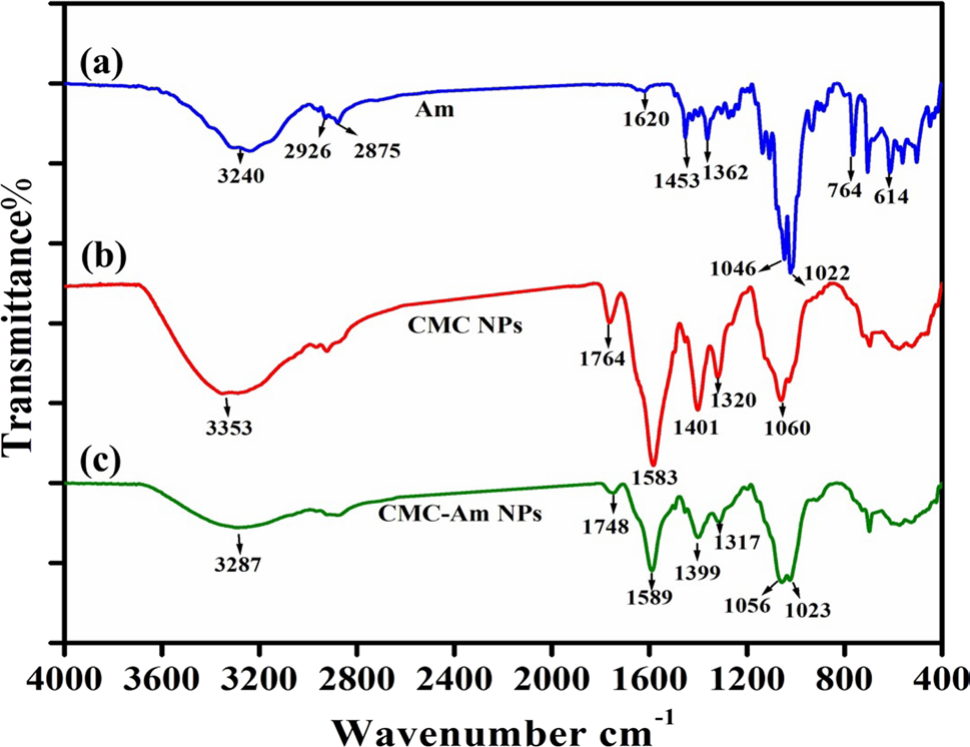
FTIR spectrum of **a** amygdalin, **b** CMC NPs and **c** CMC-Am NPs

*FTIR spectroscopy* is considered as useful technique to investigate whether there was any interaction between the CMC NPs and amygdalin during the encapsulation. Interaction was predicted by changes in the frequency of the characteristics peaks of the NPs and drug when these components are mixed. The FTIR spectra of amygdalin, CMC NPs and amygdalin-loaded CMC NPs are illustrated in Fig. 3. The amygdalin FTIR spectrum; Fig. 3a shows absorption bands at 3240 cm^−1^ attributed to O–H stretching vibrations in the glucose molecule, 2926 cm^−1^ and 2875 cm^−1^ corresponded to aromatic and aliphatic C–H stretching, respectively. The bands at 1620 and 1453 cm^−1^ corresponded to C=C stretching vibrations in the benzene ring. The bands observed at 1046 cm^−1^ and 614 cm^−1^ attributed to C–O stretching and C–H out of plane bending vibrations, respectively [20].

Figure 3b shows the basic characteristic peak of CMC NPs at 3353 cm^−1^ which corresponded to the NH_2_ and OH groups stretching vibrations. The bands at 2922 cm^−1^ and 1764 cm^−1^ corresponded to aliphatic C–H and carbonyl groups stretching, respectively. The strong bands at 1583 cm^−1^ and 1401 cm^−1^ corresponded to the N–H bending and COO^−^ stretching vibration, respectively. The bands at 1320 cm^−1^ and 1060 cm^−1^ corresponded to stretching of C–O groups [21].

In the spectrum of amygdalin-loaded CMC NPs; Fig. 3c, the characteristic bands of CMC NPs are not affected by the entrapment of amygdalin as they are slightly shifted from 1764, 1583 and 1401 cm^−1^ to 1748, 1589 and 1399 cm^−1^, respectively as shown in Table 1. Hence, there is no new chemical bond was formed between the functional group in the amygdalin and the CMC after preparing the nanoparticles. Therefore, amygdalin is entrapped physically in the CMC NPs without making any significant chemical interactions confirming the amygdalin stability and compatibility with polymer during the encapsulation process [20, 22]. Moreover, in the spectra of CMC-Am NPs, there was a reduction in the peak intensity at 1589 cm^−1^ and 1399 cm^−1^ indicating the successful encapsulation of amygdalin within CMC nanoparticles.

**Table 1:** FTIR band assignment for blank CMC NPs and amygdalin encapsulated CMC NPs (CMC-Am NPs)

CMC NPs wavenumber (cm^−1^)	CMC-Am NPs wavenumber (cm^−1^)	Band assignment
3353	3287	NH_2_ and OH groups stretching
2922	2923	Aliphatic C–H bond stretching
1764	1748	C=O carbonyl groups stretching
1583	1589	N–H bending
1401	1399	COO^−^ stretching vibration
1060	1056	Stretching of C–O groups

#### Dielectric spectroscopy

**Fig. 4:**
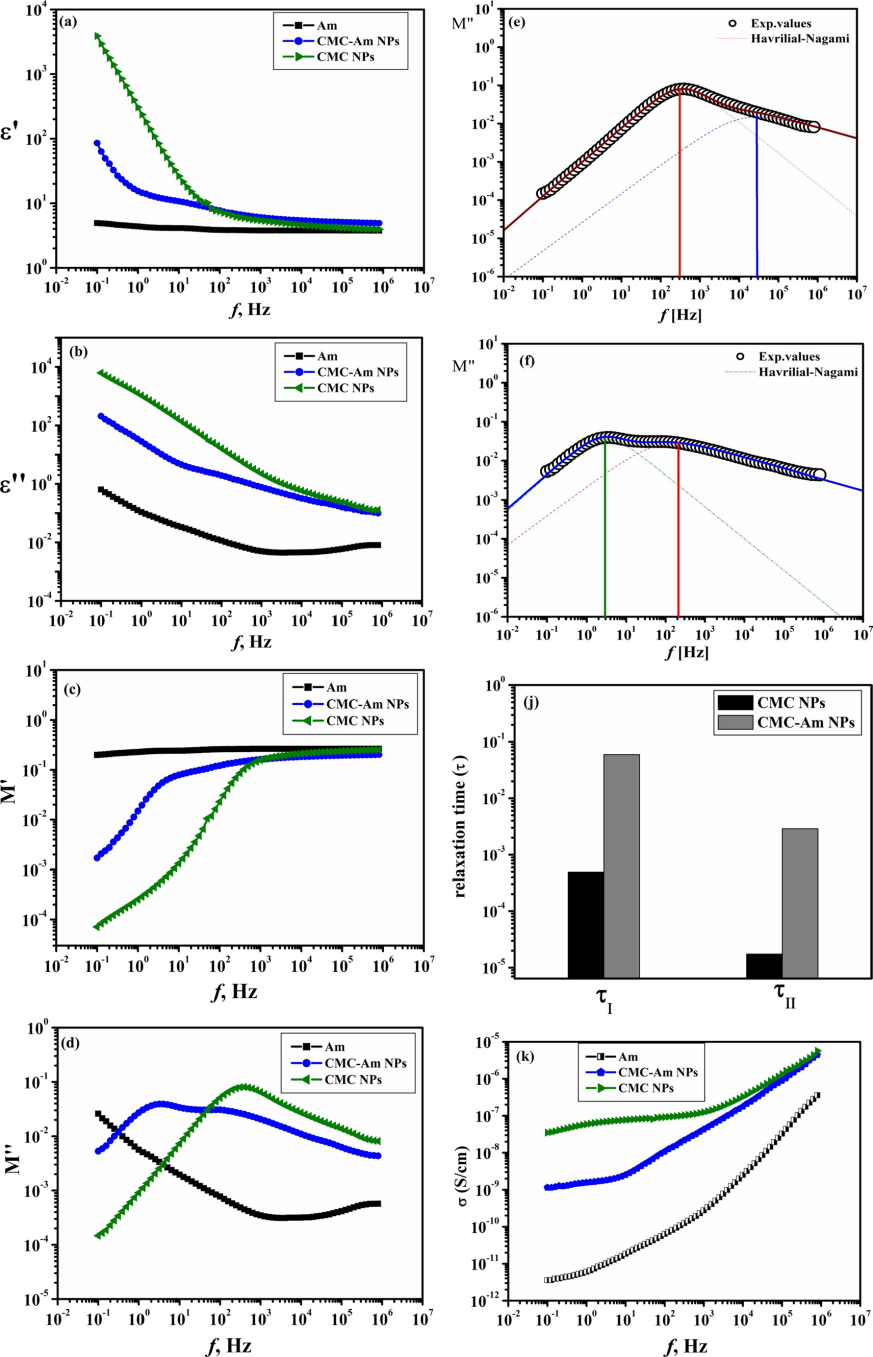
**a** Variation of the permittivity (ε′) versus frequency (*f*). **b**: Variation of the dielectric loss (ε″) versus frequency (*f*). **c**: Variation of the real part of electric modulus (M′) versus frequency (*f*). **d**: Variation of the imaginary part of electric modulus (M″) versus frequency (*f*). **e**: Example of the analysis for CMC NPs. **f**: Example of the analysis for CMC-Am NPs. **j**: Variation of the relaxation time (τ) for CMC NPs and CMC-Am NPs. **k**: Variation of the ac conductivity (σ) versus frequency (*f*)

*The dielectric parameters* were measured for amygdalin, CMC NPs as well as amygdalin-loaded CMC NPs over a broad range of frequency between 10^–2^ and 10^5^ Hz at room temperature. Figure 4a and b show the dependence of the permittivity (ε′) and dielectric loss (ε″) on the frequency for amygdalin, CMC NPs and CMC-loaded amygdalin NPs. In all samples, the permittivity (ε′) increased tremendously in the low frequency region as result of space charge effect and electrode polarization at the sample-electrode interface [23]. Then, the permittivity (ε′) values decreased as the applied alternating electric field’s frequency increased until they reached nearly constant values in the high frequency region.

Also, in Fig. 4a and b, it was noticed that the dielectric constant (ε′) and loss values (ε'') are decreased after the encapsulation of amygdalin within CMC NPs which can be attributed to some sort of interaction took place between amygdalin and CMC NPs. Comparable results were obtained by Hegazi et al. [24] who found that the values of ε′ and ε″ were reduced in the ALg-loaded propolis NPs in comparison to blank alginate NPs. Also, El Sayed et al. [25] found that the ε' value was affected by the film composition as it decreased after blending PVP with chitosan.

Furthermore, the dielectric loss (ε'') curve; Fig. 4b expressed a non-Debye response in which many relaxation processes were observed. This behavior is caused by the accumulation of free charges at the sample-electrode interface which resulted in the electrode polarization effect when an electric field is passed across the sample. At lower frequencies, the electrode polarization overlapped with further relaxation processes in addition to the term of electrical conductivity. Therefore, to suppress the conductivity and polarization effects at the electrode-sample interface, the formula of electric modulus was applied [26].

Figure 4c and d represents the real part (M') and imaginary part (M'') of electric modulus versus frequency for amygdalin, CMC NPs and CMC-Am NPs. The plot of the electrical modulus (M'') with respect to frequency (*f*) was analyzed using a multi-peak fitting program at room temperature. Examples of the analysis for CMC NPs and amygdalin-loaded CMC NPs are illustrated Fig. 4e and f which show the presence of two superimposed dielectric relaxations fitted by two Havriliak–Negami functions. In the low frequency region, the first relaxation process, was corresponded to the motion of the main polymeric chain (crankshaft type αβ), whereas the second process in the high frequency region was corresponded to the motion of the side chains (β-relaxation). It seemed that the segmental mobility of the CMC NPs chains is decreased after the encapsulation of amygdalin. The shift of the first and second relaxation peaks to lower frequencies for amygdalin-loaded CMC NPs compared to blank CMC NPs is a result of the dielectric relaxation time was increased. This means that the encapsulation of CMC NPs with amygdalin causes the chains of CMC un-flexible which reflecting some sort of interaction occurred between amygdalin and CMC NPs therefore increasing the relaxation time for this process; Fig. 4j. Similar findings were reported by El-Houssiny et al. [27] who found that the first peak of relaxation process was moved to lower frequency in the glucosamine-loaded alginate NPs relative to blank ALg NPs.

Furthermore, to confirm the above results, the electrical conductivity for amygdalin, CMC NPs and amygdalin loaded-CMC NPs was measured as function of frequency and illustrated in Fig. 4k. It was noticed that after the encapsulation of amygdalin within CMC NPs, the conductivity values are decreased compared to blank CMC NPs. This suggested that interaction between amygdalin and CMC NPs limited the ions’ electrical mobility, causing more compact structure. Similar findings were observed, showing that the crosslinking of alginate with Ca^+2^ ions caused a reduction in the conductivity values of Alg-crosslinked films relative to blank alginate films [28].

#### *Invitro* drug release experiment

**Fig. 5:**
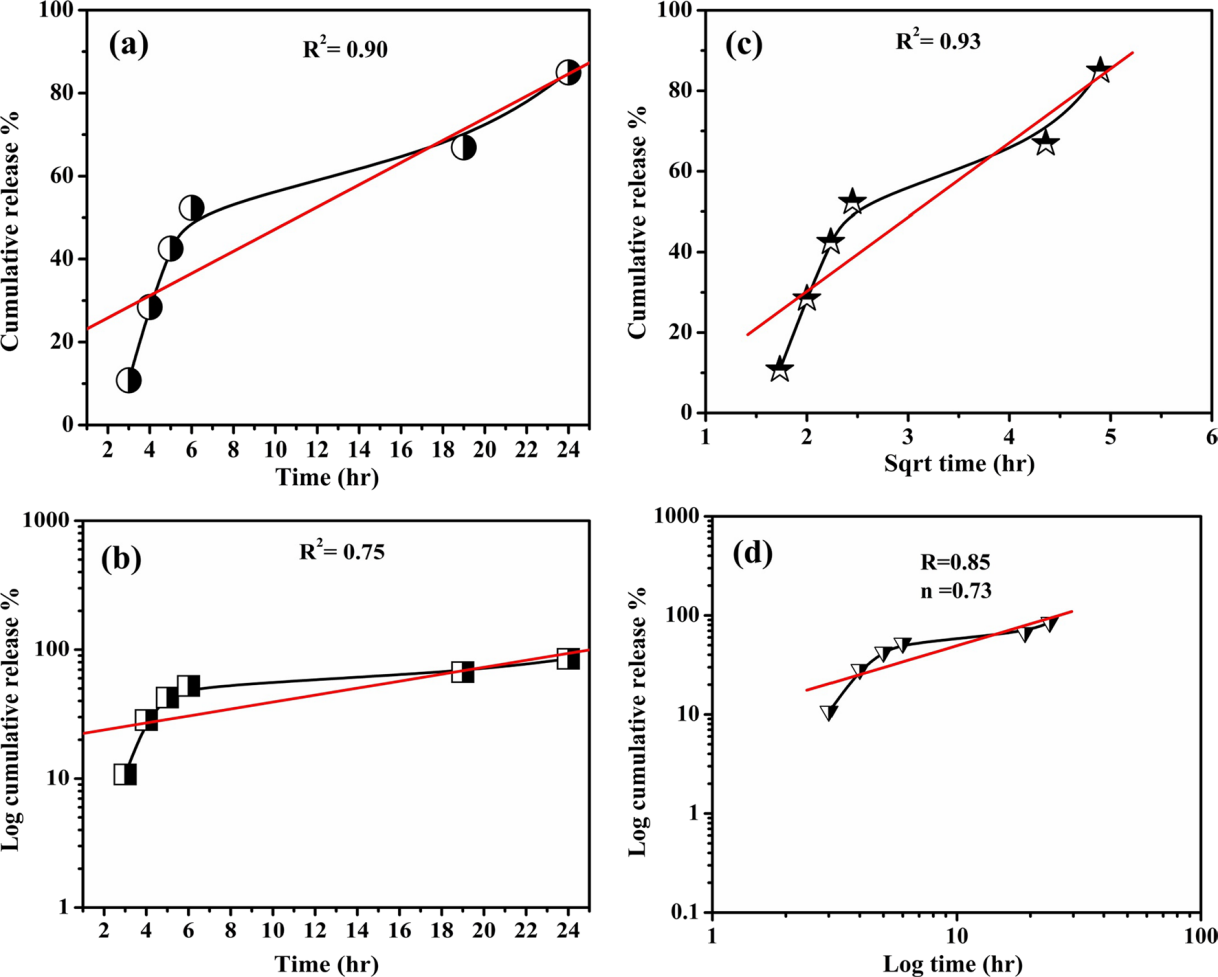
**a** Release profile of amygdalin from CMC NPs ( Zero-order kinetics). **b**: Release profile of amygdalin from CMC NPs (First-order kinetics).** c**: Release profile of amygdalin from CMC NPs (Higuchi kinetics).** d**: Release profile of amygdalin from CMC NPs (Korsmeyer-Peppas kinetics)

The amygdalin *invitro* release profile from CMC NPs in phosphate buffer solution (PBS pH 7.4) was examined as shown in Fig. 5. According to this figure, the amygdalin release from nanoparticles exhibited a gradual release profile of about 52% during the first 6 h, followed by a sustained and complete release of amygdalin up to 90% over 24 h [29]. The initial release of amygdalin is caused by the diffusion of amygdalin molecules dispersed at the CMC NPs surface during the initial incubation period. Whereas, the sustained release is attributed to those amygdalin molecules incorporated within the nanopartice network. Additionally, a larger quantity of carboxylic acid salt (COO-) was formed at a higher PBS pH 7.4, which enhanced the electrostatic repulsion between and among molecules. This in turn increased the pore size and cumulative release of amygdalin from CMC NPs. Therefore, the amygdalin *invitro* release exhibited a sustained release profile and suggested that CMC NPs can adsorb amygdalin not only on their large surface area but also in their pores.

Several release kinetic models can be applied to predict the drug release behavior mechanisms which controlled by the drug diffusion through the polymer matrix, erosion or swelling of the polymer chains [30]. The amygdalin *invitro* release profile from CMC NPs was fitted into several kinetic equations. For zero-order model release, the cumulative release (Q_t_) is plotted versus time (t); ( ${{\text{Q}}}_{{\text{t}}}={{\text{Q}}}_{0}+ k t$). For the first-order release model, log cumulative release is plotted versus time; (${\mathrm{Log Q}}_{{\text{t}}}={\mathrm{Log Q}}_{0}+kt/2.303$). For the Higuchi release model, cumulative release is plotted versus square root of time; (${{\text{Q}}}_{{\text{t}}}=\mathrm{k }{{\text{t}}}^{1/2}$). For the Korsmeyer-Peppas model, log cumulative release is plotted versus log time; ( ${{\text{M}}}_{{\text{t}}}/{{\text{M}}}_{\infty }=\mathrm{K }{{\text{t}}}^{{\text{n}}}$). Q_o_ is the initial drug release amount, K is the kinetic constant, M_t_/M_∞_ is the drug release fraction and n is the release exponent, which indicates the drug release mechanism. When the value of n less than 0.43, it suggests that the drug diffused following Fickian diffusion. However, when the value of n greater than 0.85, the mechanism of drug release is considered as non Fickian, meaning that the drug is controlled by an erosion process. While, the drug release is referred as anomalous when 0.43 < n < 0.85, and controlled by combination of erosion and diffusion.

According to the results, the drug release data for CMC-Am NPs was highest fitted by Higuchi kinetics; Fig. 5c (R^2^ = 0.94) followed by zero kinetics; Fig. 5a (R^2^ = 0.91) then first kinetics; Fig. 5b (R^2^ = 0.75). In Higuchi kinetic model, the drug release is dependent on the diffusion through the matrix based on the Fick law [31]. Also, the release of amygdalin from CMC NPs showed anomalous diffusion, which controlled by diffusion-swelling mechanism of the polymer molecular chains where the value of n is 0.73; Fig. 5d. Comparable results were obtained by Zhang et al., (2021) who found that the release of protein drug (BSA) from carboxymethyl β‑cyclodextrin/chitosan complex in phosphate buffer pH 7.4 was controlled by both swelling and diffusion where the value of n lies between 0.5 and 1 [32]. Also, Jayakumar et al. [33] found that the release of indomethacin from N-carboxymethyl chitosan beads may be controlled by swelling followed by diffusion. Moreover, Yadav et al. [34] found that the value of n was lies between 0.5 and 0.7, which indicates the drug release from the carboxymethyl chitosan hydrogels followed anomalous mechanism.

#### *InVitro* anticancer activity studies (MTT-ASSAY)

Amygdalin is a cyanogenic organic molecule that is commonly utilized in traditional medicine [35]. However, the enzymatic breakdown of the amygdalin produces hydrogen cyanide, which is toxic to live cells. As a result, the use of this molecule still presents a number of difficulties and need additional research. In addition, some case report studies have revealed that given high dosage of this compound or oral administration can cause cyanide poisoning in patients which results from increased intestinal enzymatic degradation and hydrogen cyanide release compared with the intravenous injection. Therefore, to address these challenges, nano-formulations of amygdalin have been investigated to elevate bioavailability, increase therapeutic effects, reduce the effective dose of amygdalin, enhance the anticancer effects of amygdalin and decrease dose dependent side effects. Hence, the main objective of this study was to minimize the effective dose of amygdalin and enhance its impact on MCF-7 and HCT-116 cancer cell lines by nanoformulating it using CMC NPs.

**Fig. 6:**
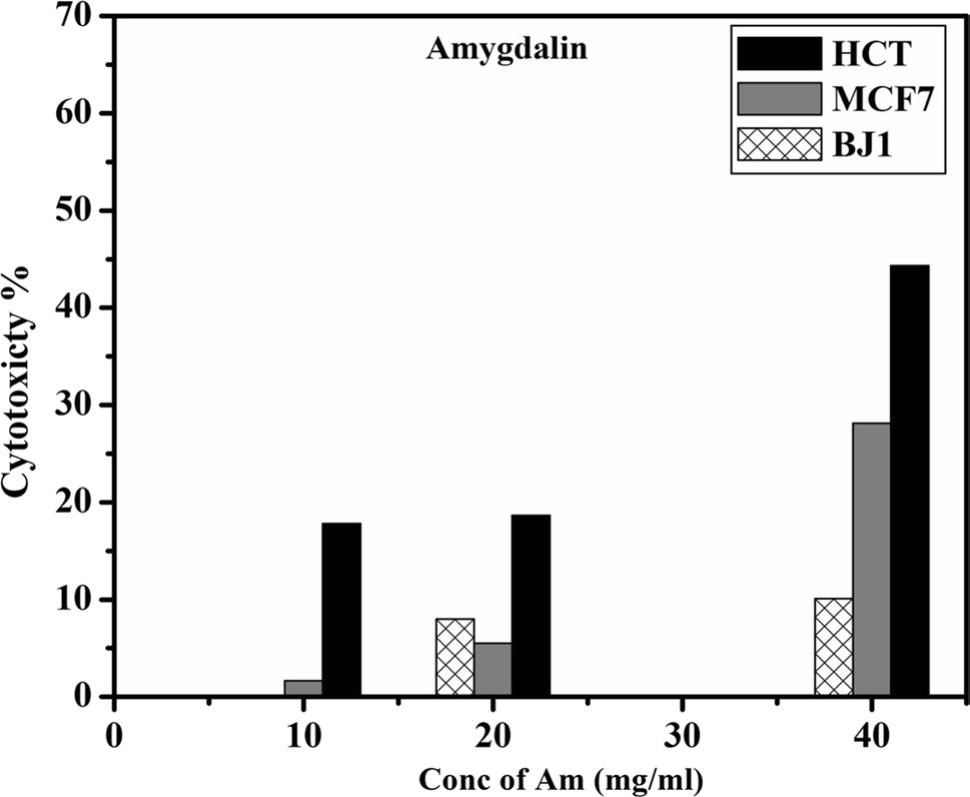
Cytotoxicity of amygdalin against colon carcinoma (HCT-116), breast carcinoma (MCf-7), and normal skin fibroblast cells (BJ1)

The cytotoxicity effects of free drug (amygdalin) against HCT-116, MCF-7 and skin fibroblast normal cells (BJ1) are shown in Fig. 6. The amygdalin cytotoxcity on HCT-116 cells was found to be 17.8%, 18.66% and 44.33% at concentration 10, 20 and 40 mg/ml, respectively. The amygdalin cytotoxcity on MCF-7 cells was calculated to be 1.66%, 5.5% and 28.13% at concentration 10, 20 and 40 mg/ml, respectively. While, adding the same concentration to normal skin firoblast cells (BJ1) did not show any significant effect on cytotoxity of cells about 1%, 8% and 10%.

Amygdalin was broken down into sugar, hydrocyanic acid which is a toxic compound, and benzaldehyde by the activity of enzyme b-glucosidase which present only in the cancer cells. The presence of β- glucosidase enzyme in the cancer cells may be responsible for the reduced viability of the colon HCT-116 and breast MCF-7 cancer cell lines which release the cyanide ions from the amygdalin breakdown. Cyanide ion can bind to cytochrome C oxidase, an enzyme located within the mitochondria, which inhibits cellular respiration (electron transport chain) [36]. Cyanide ions-induced reactive oxygen species (ROS) overproduction, and led to the subsequent benzaldehyde over production, which may trigger protein oxidation, causing increased lipid peroxidation levels and oxidative stress [37]. This mechanism may be associated with the anticancer effects of amygdalin by forming several reactive oxygen species which, interfere with respiration and DNA synthesis, obstruct cell nutrition and lead to lysis. Therefore, the anticancer effect of amygdalin, compared with control, are the main outcomes of high levels of ROS induced by cyanide ions released. Furthermore, in numerous clinical trials, benzaldehyde is shown to be an anti-cancer medication [38]. Subsequently, the cyanide and benzaldehyde that are released target cancer cells by creating a lethal toxin [39]. Accordingly, it is proposed that amygdalin kills cancer cells by the synergistic activities of its two main metabolites, benzaldehyde and hydrocyanic acid.

While, normal cells contain the mitochondrial enzyme ''rhodanese'' (thiosulfate sulfur transferase) which is capable of neutralizing the harmful compounds (hydrogen cyanide) and (benzaldahyde) in amygdalin to thiocyanate (rhodanide) and benzoic acid, both of which are non-toxic compounds [38]. The enzyme rhodanese removes sulfur from the amino acid ''cysteine'' and combines it with hydrocyanic acid to form the relatively safe chemical ''thiocyanate'', which is 200 times less toxic than cyanide. In contrast, because cancer cells lack the enzyme rhodanese, amygdalin that is given to them doesn't neutralized to rhodanide and benzoic acid. It was observed that tissues taken from several malignancies, such as those of the stomach, esophagus, abdominal wall, uterus, and breast, had 100–3600% more β-D-glucosidase activity than adjacent normal tissues. Therefore, cancer cells contain two distinguishing feature, high level of enzyme ''β-D-glucosidase'', and an insufficiency of detoxifying enzyme ''rhodanese''. If any hydrocyanic acid leaks from malignant cells, it is denatured by the rhodanese enzyme in the surrounding normal healthy cells. This gives rise to rational explanations for why amygdalin preferentially targets malignant cells while remaining harmless to normal cells. Therefore, in our results, the absence of rhodanese enzyme in cancer cells and presence of β- glucosidase enzyme may be responsible for the cytotoxcicity of MCF-7 and HCT-116 cell lines while maintaining the viability of normal cell line BJ1.

**Fig. 7:**
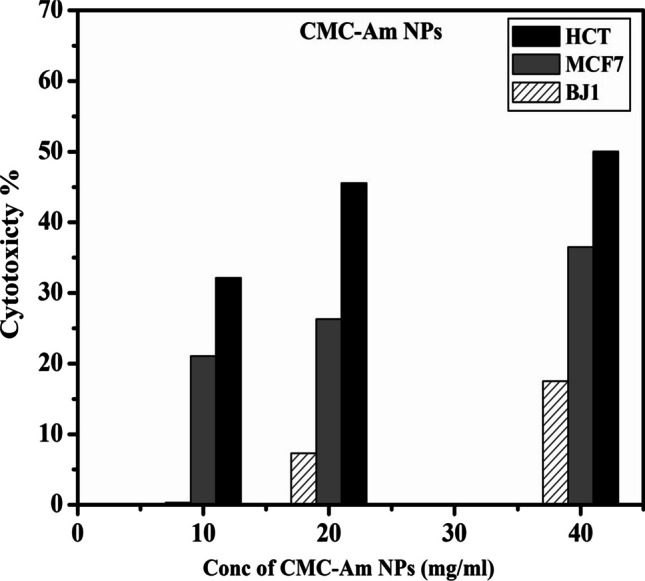
Cytotoxicity of CMC-Am NPs against colon carcinoma (HCT-116), breast carcinoma (MCf-7), and normal skin fibroblast cells (BJ1)

On the other hand, the cytotoxicity effects of amygdalin-loaded CMC NPs on HCT-116, MCF-7 and skin fibroblast normal cells (BJ1) are shown in Fig. 7. It was found that the cytotoxicty was enhanced significantly in a dose dependent of the CMC-Am NPs. The percentage of increasing cytotoxicity was 32.13%, 45.56% and 50.03% at concentration 10, 20 and 40 mg/ml, respectively on colon HCT-116 cell line. While, for breast MCF7 cells, the percentage of increasing cytotoxicity was 21.08%, 26.29% and 36.5% at concentration 10, 20 and 40 mg/ml, respectively. Furthermore, when the CMC-Am NPs were evaluated on skin fibroblast normal cells (BJ1), the cell cytotoxicity percentage was determined about 0.3%, 7.3% and 17% at concentration 10, 20 and 40 mg/ml, respectively indicating that the CMC-Am NPs was biocompatible with the normal BJ1 cell.

According to the above results, it was observed that amygdalin-loaded CMC NPs have enhanced anticancerous effect as it shown cytotoxicity about 50.03% and 36.5% at concentration 40 mg/ml on HCT-116 and MCF-7 cells, respectively compared to 44.33% and 28.13% for free amygdalin. Consequently, in our study the current CMC-Am nanoformuation reduced the effective dose of amygdalin, provided prolonged release, increased the therapeutic benefits of amygdalin, improved amygdalin anticancerous efficacy compared to free amygdalin. The enhanced anti-cancerous effect of amygdalin encapsulated CMC NPs system against colon HCT-116 and breast MCF-7 cancer cell lines compared to free amygdalin are due to the CMC-Am NPs have small particle size, provide sustained drug release over time, can focus amygdalin delivery to cancer cells, increase drug permeability and hence the CMC-Am NPs can be more effective in cytotoxicity suggesting greater cellular uptake of the CMC nanoparticles.

These results are inconsistent with the study reported by Mosayyebi et al. [12] who found that b-Cyclodextrin-amygdalin nanoparticles enhanced the cell cytotoxcity of MCF-7 cells more than amygdalin. Furthermore, our findings are inconsistent with those of Sohail et al. [20], who demonstrated that the chitosan-alginate complex NPs encapsulated amygdalin had a stronger cytotoxic effect on H1299 cells in a dose-dependent manner than free amygdalin, indicating a larger cellular uptake of the NPs. Therefore, our findings indicated that CMC-loaded amygdalin NPs are promising carrier for drug administration against HCT-116 and MCF-7 cancer cells, with negligible cytotoxicity in fibroblast normal cells (BJ1).

## Conclusions

In this study, amygdalin-loaded CMC NPs with a high colloidal stability (-43.5 mV) and small particle size in the nano-range of 129 nm were effectively synthesized using the ionic gelation method. The current study offers CMC NPs as an excellent drug delivery vehicle for amygdalin, providing prolonged release and enhancing amygdalin efficacy and delivery. When compared to the free drug (amygdalin), the CMC-Am NPs had significant cytotoxic action against both HCT-116 and MCF-7 cell lines and were harmless for fibroblast normal cells (BJ1). As a result, CMC-Am NPs are regarded as one of the most promising choices for cancer cells targeting. This promotes further *invivo* testing of amygdalin-loaded CMC NPs on cancer tumor models.

## Data Availability

All information created or analyzed during the present study are included in the manuscript.
